# Dichloridobis(2-methyl­quinolin-8-olato-κ^2^ *N*,*O*)tin(IV)

**DOI:** 10.1107/S1600536809020340

**Published:** 2009-06-06

**Authors:** Kong Mun Lo, Seik Weng Ng

**Affiliations:** a Department of Chemistry, University of Malaya, 50603 Kuala Lumpur, Malaysia

## Abstract

The bis-chelated Sn atom in the title compound, [Sn(C_10_H_8_NO)_2_Cl_2_], exists in a distorted *cis*-Cl_2_,*cis*-N_2_,*trans*-O_2_ octa­hedral environment.

## Related literature

For the crystal structure of dichloridobis(8-oxidoquinoline), see: Archer *et al.* (1987 ▶).
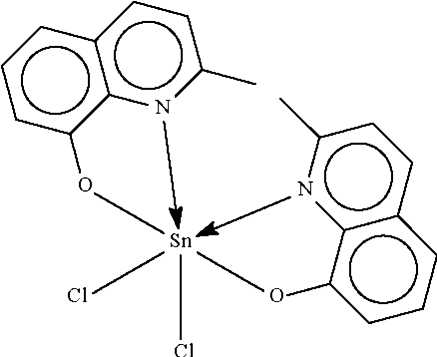


## Experimental

### 

#### Crystal data

[Sn(C_10_H_8_NO)_2_Cl_2_]
*M* *_r_* = 505.94
Triclinic, 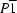

*a* = 7.9651 (1) Å
*b* = 9.6336 (1) Å
*c* = 12.8337 (2) Å
α = 94.599 (1)°
β = 90.262 (1)°
γ = 109.236 (1)°
*V* = 926.29 (2) Å^3^
*Z* = 2
Mo *K*α radiation
μ = 1.69 mm^−1^
*T* = 133 K
0.20 × 0.10 × 0.05 mm

#### Data collection

Bruker SMART APEX diffractometer
Absorption correction: multi-scan (*SADABS*; Sheldrick, 1996 ▶) *T*_min_ = 0.729, *T*_max_ = 0.920
7670 measured reflections
4189 independent reflections
3905 reflections with *I* > 2σ(*I*)
*R*_int_ = 0.015

#### Refinement

*R*[*F* ^2^ > 2σ(*F* ^2^)] = 0.023
*wR*(*F* ^2^) = 0.060
*S* = 1.03
4189 reflections
246 parameters
H-atom parameters constrained
Δρ_max_ = 0.79 e Å^−3^
Δρ_min_ = −0.34 e Å^−3^

### 

Data collection: *APEX2* (Bruker, 2007 ▶); cell refinement: *SAINT* (Bruker, 2007 ▶); data reduction: *SAINT*; program(s) used to solve structure: *SHELXS97* (Sheldrick, 2008 ▶); program(s) used to refine structure: *SHELXL97* (Sheldrick, 2008 ▶); molecular graphics: *X-SEED* (Barbour, 2001 ▶); software used to prepare material for publication: *publCIF* (Westrip, 2009 ▶).

## Supplementary Material

Crystal structure: contains datablocks global, I. DOI: 10.1107/S1600536809020340/tk2467sup1.cif

Structure factors: contains datablocks I. DOI: 10.1107/S1600536809020340/tk2467Isup2.hkl

Additional supplementary materials:  crystallographic information; 3D view; checkCIF report

## Figures and Tables

**Fig. 1.**
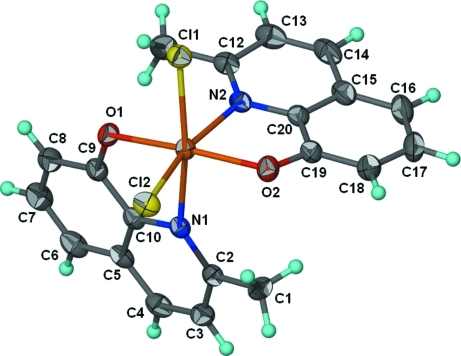
Thermal ellipsoid plot (Barbour, 2001) of SnCl2(C10H8NO)2 at the 70% probability level. Hydrogen atoms are drawn as spheres of arbitrary radius.

## supplementary crystallographic information

### Experimental

Di(*p*-chlorobenzyl)tin dichloride (0.44 g, 1 mmol) and 8-hydroxyquinaldine (0.16 g, 1 mmol) were dissolved in chloroform (100 ml) and the solution was heated for 1 hour. Slow evaporation of the filtrate gave yellow crystals. The organic groups at tin were probabaly cleaved by the heterocycle in the reaction.

### Refinement

Hydrogen atoms were placed at calculated positions (C–H 0.95–0.98 Å) and were treated as riding on their parent atoms, with *U*(H) set to 1.2–1.5 times *U*_eq_(C).

### Crystal data

**Table:**

[Sn(C_10_H_8_NO)_2_Cl_2_]	*Z* = 2
*M**_r_* = 505.94	*F*(000) = 500
Triclinic, *P*1	*D*_x_ = 1.814 Mg m^−^^3^
Hall symbol: -P 1	Mo *K*α radiation, λ = 0.71073 Å
*a* = 7.9651 (1) Å	Cell parameters from 9941 reflections
*b* = 9.6336 (1) Å	θ = 2.4–28.2°
*c* = 12.8337 (2) Å	µ = 1.69 mm^−^^1^
α = 94.599 (1)°	*T* = 133 K
β = 90.262 (1)°	Prism, pale yellow
γ = 109.236 (1)°	0.20 × 0.10 × 0.05 mm
*V* = 926.29 (2) Å^3^	

### Data collection

**Table:**

Bruker SMART APEX diffractometer	4189 independent reflections
Radiation source: fine-focus sealed tube	3905 reflections with *I* > 2σ(*I*)
graphite	*R*_int_ = 0.015
ω scans	θ_max_ = 27.5°, θ_min_ = 1.6°
Absorption correction: multi-scan (*SADABS*; Sheldrick, 1996)	*h* = −10→10
*T*_min_ = 0.729, *T*_max_ = 0.920	*k* = −12→12
7670 measured reflections	*l* = −16→16

### Refinement

**Table:**

Refinement on *F*^2^	Primary atom site location: structure-invariant direct methods
Least-squares matrix: full	Secondary atom site location: difference Fourier map
*R*[*F*^2^ > 2σ(*F*^2^)] = 0.023	Hydrogen site location: inferred from neighbouring sites
*wR*(*F*^2^) = 0.060	H-atom parameters constrained
*S* = 1.03	*w* = 1/[σ^2^(*F*_o_^2^) + (0.0346*P*)^2^ + 0.4719*P*] where *P* = (*F*_o_^2^ + 2*F*_c_^2^)/3
4189 reflections	(Δ/σ)_max_ = 0.001
246 parameters	Δρ_max_ = 0.79 e Å^−^^3^
0 restraints	Δρ_min_ = −0.34 e Å^−^^3^

### Fractional atomic coordinates and isotropic or equivalent isotropic displacement parameters (Å^2^)

**Table:**

	*x*	*y*	*z*	*U*_iso_*/*U*_eq_	
Sn1	0.276677 (18)	0.727420 (15)	0.279378 (11)	0.01746 (6)	
Cl1	0.03285 (7)	0.77602 (6)	0.20001 (4)	0.02382 (12)	
Cl2	0.24196 (8)	0.82902 (6)	0.44903 (4)	0.02532 (12)	
N1	0.4783 (2)	0.6315 (2)	0.34153 (14)	0.0183 (4)	
N2	0.3536 (2)	0.6733 (2)	0.11290 (15)	0.0200 (4)	
O1	0.1236 (2)	0.51786 (17)	0.29867 (13)	0.0221 (3)	
O2	0.4661 (2)	0.91742 (17)	0.25039 (13)	0.0240 (3)	
C1	0.7366 (3)	0.8568 (3)	0.37939 (19)	0.0250 (5)	
H1A	0.6522	0.9047	0.4057	0.038*	
H1B	0.7746	0.8885	0.3101	0.038*	
H1C	0.8406	0.8847	0.4275	0.038*	
C2	0.6490 (3)	0.6930 (3)	0.37157 (17)	0.0200 (4)	
C3	0.7488 (3)	0.6025 (3)	0.39744 (19)	0.0247 (5)	
H3	0.8709	0.6470	0.4177	0.030*	
C4	0.6739 (3)	0.4537 (3)	0.39396 (19)	0.0260 (5)	
H4	0.7444	0.3948	0.4093	0.031*	
C5	0.4912 (3)	0.3865 (3)	0.36755 (17)	0.0225 (5)	
C6	0.3986 (4)	0.2331 (3)	0.36603 (19)	0.0283 (5)	
H6	0.4603	0.1673	0.3807	0.034*	
C7	0.2184 (4)	0.1802 (3)	0.3431 (2)	0.0311 (6)	
H7	0.1567	0.0772	0.3419	0.037*	
C8	0.1239 (3)	0.2753 (3)	0.32133 (19)	0.0272 (5)	
H8	−0.0009	0.2360	0.3073	0.033*	
C9	0.2103 (3)	0.4248 (2)	0.32009 (17)	0.0208 (4)	
C10	0.3970 (3)	0.4819 (2)	0.34239 (17)	0.0192 (4)	
C11	0.1273 (3)	0.4305 (3)	0.0696 (2)	0.0285 (5)	
H11A	0.0424	0.4658	0.1094	0.043*	
H11B	0.1706	0.3684	0.1120	0.043*	
H11C	0.0685	0.3729	0.0053	0.043*	
C12	0.2808 (3)	0.5597 (3)	0.04230 (18)	0.0228 (5)	
C13	0.3471 (4)	0.5605 (3)	−0.0607 (2)	0.0302 (5)	
H13	0.2915	0.4812	−0.1117	0.036*	
C14	0.4896 (4)	0.6746 (3)	−0.0858 (2)	0.0313 (6)	
H14	0.5352	0.6729	−0.1540	0.038*	
C15	0.5713 (3)	0.7955 (3)	−0.01254 (19)	0.0269 (5)	
C16	0.7162 (3)	0.9198 (3)	−0.0338 (2)	0.0348 (6)	
H16	0.7712	0.9228	−0.0995	0.042*	
C17	0.7769 (4)	1.0357 (3)	0.0405 (2)	0.0374 (7)	
H17	0.8772	1.1177	0.0265	0.045*	
C18	0.6947 (3)	1.0370 (3)	0.1376 (2)	0.0311 (6)	
H18	0.7378	1.1204	0.1871	0.037*	
C19	0.5515 (3)	0.9171 (3)	0.16080 (19)	0.0238 (5)	
C20	0.4932 (3)	0.7921 (3)	0.08655 (18)	0.0212 (5)	

### Atomic displacement parameters (Å^2^)

**Table:**

	*U*^11^	*U*^22^	*U*^33^	*U*^12^	*U*^13^	*U*^23^
Sn1	0.01921 (8)	0.01482 (8)	0.01839 (9)	0.00572 (6)	0.00134 (6)	0.00120 (6)
Cl1	0.0241 (3)	0.0261 (3)	0.0234 (3)	0.0112 (2)	0.0001 (2)	0.0025 (2)
Cl2	0.0310 (3)	0.0255 (3)	0.0213 (3)	0.0128 (2)	0.0006 (2)	−0.0018 (2)
N1	0.0213 (9)	0.0184 (9)	0.0158 (9)	0.0071 (7)	0.0017 (7)	0.0015 (7)
N2	0.0210 (9)	0.0214 (9)	0.0193 (9)	0.0091 (8)	0.0002 (7)	0.0034 (8)
O1	0.0194 (7)	0.0183 (8)	0.0275 (9)	0.0041 (6)	0.0000 (6)	0.0045 (7)
O2	0.0267 (8)	0.0169 (8)	0.0258 (9)	0.0037 (6)	0.0018 (7)	0.0016 (7)
C1	0.0209 (10)	0.0239 (12)	0.0271 (12)	0.0038 (9)	−0.0021 (9)	−0.0004 (10)
C2	0.0215 (10)	0.0230 (11)	0.0152 (10)	0.0067 (9)	0.0024 (8)	0.0019 (9)
C3	0.0206 (10)	0.0332 (13)	0.0220 (12)	0.0110 (10)	0.0017 (9)	0.0042 (10)
C4	0.0322 (12)	0.0309 (13)	0.0214 (12)	0.0187 (11)	0.0019 (9)	0.0048 (10)
C5	0.0310 (12)	0.0241 (11)	0.0150 (11)	0.0124 (10)	0.0016 (9)	0.0026 (9)
C6	0.0436 (14)	0.0219 (12)	0.0236 (12)	0.0161 (11)	−0.0004 (10)	0.0035 (10)
C7	0.0463 (15)	0.0151 (11)	0.0286 (13)	0.0058 (10)	−0.0042 (11)	0.0019 (10)
C8	0.0304 (12)	0.0218 (12)	0.0260 (13)	0.0036 (10)	−0.0031 (10)	0.0040 (10)
C9	0.0265 (11)	0.0172 (10)	0.0182 (11)	0.0064 (9)	0.0004 (9)	0.0020 (9)
C10	0.0262 (11)	0.0173 (10)	0.0144 (10)	0.0080 (9)	0.0014 (8)	0.0005 (8)
C11	0.0342 (13)	0.0235 (12)	0.0256 (13)	0.0074 (10)	−0.0039 (10)	−0.0018 (10)
C12	0.0259 (11)	0.0239 (11)	0.0216 (12)	0.0123 (9)	−0.0006 (9)	0.0010 (9)
C13	0.0362 (13)	0.0364 (14)	0.0233 (12)	0.0198 (12)	−0.0007 (10)	−0.0004 (11)
C14	0.0365 (13)	0.0439 (15)	0.0199 (12)	0.0213 (12)	0.0050 (10)	0.0065 (11)
C15	0.0265 (12)	0.0349 (14)	0.0241 (12)	0.0150 (10)	0.0038 (10)	0.0088 (11)
C16	0.0294 (13)	0.0446 (16)	0.0329 (15)	0.0120 (12)	0.0106 (11)	0.0184 (13)
C17	0.0258 (12)	0.0406 (16)	0.0446 (17)	0.0049 (11)	0.0063 (11)	0.0231 (14)
C18	0.0285 (12)	0.0271 (13)	0.0349 (14)	0.0035 (10)	−0.0028 (11)	0.0110 (11)
C19	0.0219 (10)	0.0247 (12)	0.0257 (12)	0.0076 (9)	0.0000 (9)	0.0090 (10)
C20	0.0194 (10)	0.0242 (11)	0.0223 (12)	0.0091 (9)	0.0005 (8)	0.0073 (9)

### Geometric parameters (Å, °)

**Table:**

Sn1—O2	2.0149 (16)	C6—H6	0.9500
Sn1—O1	2.0211 (16)	C7—C8	1.406 (3)
Sn1—N1	2.2703 (18)	C7—H7	0.9500
Sn1—N2	2.2925 (19)	C8—C9	1.379 (3)
Sn1—Cl2	2.3700 (6)	C8—H8	0.9500
Sn1—Cl1	2.3846 (5)	C9—C10	1.424 (3)
N1—C2	1.332 (3)	C11—C12	1.495 (3)
N1—C10	1.373 (3)	C11—H11A	0.9800
N2—C12	1.332 (3)	C11—H11B	0.9800
N2—C20	1.374 (3)	C11—H11C	0.9800
O1—C9	1.342 (3)	C12—C13	1.425 (3)
O2—C19	1.339 (3)	C13—C14	1.356 (4)
C1—C2	1.496 (3)	C13—H13	0.9500
C1—H1A	0.9800	C14—C15	1.408 (4)
C1—H1B	0.9800	C14—H14	0.9500
C1—H1C	0.9800	C15—C16	1.409 (4)
C2—C3	1.415 (3)	C15—C20	1.416 (3)
C3—C4	1.356 (4)	C16—C17	1.364 (4)
C3—H3	0.9500	C16—H16	0.9500
C4—C5	1.411 (3)	C17—C18	1.411 (4)
C4—H4	0.9500	C17—H17	0.9500
C5—C6	1.416 (3)	C18—C19	1.384 (3)
C5—C10	1.418 (3)	C18—H18	0.9500
C6—C7	1.377 (4)	C19—C20	1.420 (3)
			
O2—Sn1—O1	168.37 (6)	C6—C7—H7	119.3
O2—Sn1—N1	92.76 (7)	C8—C7—H7	119.3
O1—Sn1—N1	78.04 (6)	C9—C8—C7	120.7 (2)
O2—Sn1—N2	77.90 (7)	C9—C8—H8	119.7
O1—Sn1—N2	94.63 (7)	C7—C8—H8	119.7
N1—Sn1—N2	88.77 (6)	O1—C9—C8	121.9 (2)
O2—Sn1—Cl2	90.74 (5)	O1—C9—C10	119.2 (2)
O1—Sn1—Cl2	96.53 (5)	C8—C9—C10	118.9 (2)
N1—Sn1—Cl2	91.39 (5)	N1—C10—C5	122.3 (2)
N2—Sn1—Cl2	168.62 (5)	N1—C10—C9	117.31 (19)
O2—Sn1—Cl1	97.04 (5)	C5—C10—C9	120.4 (2)
O1—Sn1—Cl1	91.31 (5)	C12—C11—H11A	109.5
N1—Sn1—Cl1	167.95 (5)	C12—C11—H11B	109.5
N2—Sn1—Cl1	86.47 (5)	H11A—C11—H11B	109.5
Cl2—Sn1—Cl1	95.46 (2)	C12—C11—H11C	109.5
C2—N1—C10	119.95 (19)	H11A—C11—H11C	109.5
C2—N1—Sn1	132.09 (15)	H11B—C11—H11C	109.5
C10—N1—Sn1	107.93 (14)	N2—C12—C13	120.3 (2)
C12—N2—C20	120.0 (2)	N2—C12—C11	120.5 (2)
C12—N2—Sn1	132.03 (15)	C13—C12—C11	119.2 (2)
C20—N2—Sn1	107.76 (15)	C14—C13—C12	120.0 (3)
C9—O1—Sn1	116.18 (13)	C14—C13—H13	120.0
C19—O2—Sn1	116.82 (15)	C12—C13—H13	120.0
C2—C1—H1A	109.5	C13—C14—C15	121.2 (2)
C2—C1—H1B	109.5	C13—C14—H14	119.4
H1A—C1—H1B	109.5	C15—C14—H14	119.4
C2—C1—H1C	109.5	C16—C15—C20	119.3 (2)
H1A—C1—H1C	109.5	C16—C15—C14	124.4 (2)
H1B—C1—H1C	109.5	C20—C15—C14	116.2 (2)
N1—C2—C3	119.7 (2)	C17—C16—C15	119.5 (2)
N1—C2—C1	120.9 (2)	C17—C16—H16	120.2
C3—C2—C1	119.4 (2)	C15—C16—H16	120.2
C4—C3—C2	121.4 (2)	C16—C17—C18	121.7 (2)
C4—C3—H3	119.3	C16—C17—H17	119.1
C2—C3—H3	119.3	C18—C17—H17	119.1
C3—C4—C5	119.9 (2)	C19—C18—C17	120.2 (3)
C3—C4—H4	120.0	C19—C18—H18	119.9
C5—C4—H4	120.0	C17—C18—H18	119.9
C4—C5—C6	124.3 (2)	O2—C19—C18	121.6 (2)
C4—C5—C10	116.5 (2)	O2—C19—C20	119.7 (2)
C6—C5—C10	119.2 (2)	C18—C19—C20	118.7 (2)
C7—C6—C5	119.4 (2)	N2—C20—C15	122.2 (2)
C7—C6—H6	120.3	N2—C20—C19	117.4 (2)
C5—C6—H6	120.3	C15—C20—C19	120.3 (2)
C6—C7—C8	121.4 (2)		
			
O2—Sn1—N1—C2	14.7 (2)	Sn1—O1—C9—C10	8.9 (3)
O1—Sn1—N1—C2	−172.5 (2)	C7—C8—C9—O1	179.4 (2)
N2—Sn1—N1—C2	92.5 (2)	C7—C8—C9—C10	−0.7 (4)
Cl2—Sn1—N1—C2	−76.09 (19)	C2—N1—C10—C5	−4.4 (3)
Cl1—Sn1—N1—C2	159.21 (17)	Sn1—N1—C10—C5	173.84 (17)
O2—Sn1—N1—C10	−163.29 (14)	C2—N1—C10—C9	173.6 (2)
O1—Sn1—N1—C10	9.52 (14)	Sn1—N1—C10—C9	−8.2 (2)
N2—Sn1—N1—C10	−85.47 (14)	C4—C5—C10—N1	1.2 (3)
Cl2—Sn1—N1—C10	105.91 (13)	C6—C5—C10—N1	−179.5 (2)
Cl1—Sn1—N1—C10	−18.8 (3)	C4—C5—C10—C9	−176.7 (2)
O2—Sn1—N2—C12	−170.0 (2)	C6—C5—C10—C9	2.6 (3)
O1—Sn1—N2—C12	19.05 (19)	O1—C9—C10—N1	0.5 (3)
N1—Sn1—N2—C12	96.95 (19)	C8—C9—C10—N1	−179.3 (2)
Cl2—Sn1—N2—C12	−172.14 (17)	O1—C9—C10—C5	178.6 (2)
Cl1—Sn1—N2—C12	−71.98 (19)	C8—C9—C10—C5	−1.3 (3)
O2—Sn1—N2—C20	4.49 (13)	C20—N2—C12—C13	0.0 (3)
O1—Sn1—N2—C20	−166.49 (13)	Sn1—N2—C12—C13	173.93 (16)
N1—Sn1—N2—C20	−88.60 (14)	C20—N2—C12—C11	−179.3 (2)
Cl2—Sn1—N2—C20	2.3 (3)	Sn1—N2—C12—C11	−5.4 (3)
Cl1—Sn1—N2—C20	102.47 (13)	N2—C12—C13—C14	2.7 (3)
O2—Sn1—O1—C9	28.5 (4)	C11—C12—C13—C14	−178.0 (2)
N1—Sn1—O1—C9	−9.83 (15)	C12—C13—C14—C15	−1.8 (4)
N2—Sn1—O1—C9	77.92 (16)	C13—C14—C15—C16	−178.1 (2)
Cl2—Sn1—O1—C9	−99.87 (15)	C13—C14—C15—C20	−1.6 (3)
Cl1—Sn1—O1—C9	164.49 (15)	C20—C15—C16—C17	−1.1 (4)
O1—Sn1—O2—C19	44.7 (4)	C14—C15—C16—C17	175.2 (2)
N1—Sn1—O2—C19	82.07 (16)	C15—C16—C17—C18	−2.1 (4)
N2—Sn1—O2—C19	−6.07 (15)	C16—C17—C18—C19	1.8 (4)
Cl2—Sn1—O2—C19	173.50 (15)	Sn1—O2—C19—C18	−173.95 (17)
Cl1—Sn1—O2—C19	−90.91 (15)	Sn1—O2—C19—C20	7.0 (3)
C10—N1—C2—C3	4.3 (3)	C17—C18—C19—O2	−177.2 (2)
Sn1—N1—C2—C3	−173.52 (15)	C17—C18—C19—C20	1.9 (3)
C10—N1—C2—C1	−175.1 (2)	C12—N2—C20—C15	−3.7 (3)
Sn1—N1—C2—C1	7.1 (3)	Sn1—N2—C20—C15	−178.95 (17)
N1—C2—C3—C4	−1.0 (3)	C12—N2—C20—C19	172.74 (19)
C1—C2—C3—C4	178.4 (2)	Sn1—N2—C20—C19	−2.5 (2)
C2—C3—C4—C5	−2.2 (4)	C16—C15—C20—N2	−178.9 (2)
C3—C4—C5—C6	−177.2 (2)	C14—C15—C20—N2	4.4 (3)
C3—C4—C5—C10	2.1 (3)	C16—C15—C20—C19	4.7 (3)
C4—C5—C6—C7	177.4 (2)	C14—C15—C20—C19	−171.9 (2)
C10—C5—C6—C7	−1.8 (3)	O2—C19—C20—N2	−2.5 (3)
C5—C6—C7—C8	−0.2 (4)	C18—C19—C20—N2	178.4 (2)
C6—C7—C8—C9	1.5 (4)	O2—C19—C20—C15	174.04 (19)
Sn1—O1—C9—C8	−171.26 (18)	C18—C19—C20—C15	−5.1 (3)
